# Finite element analysis of lateral pressure variations in square steel silos considering parametric impacts

**DOI:** 10.1038/s41598-025-20867-0

**Published:** 2025-10-01

**Authors:** Alhussein Hilal, Abdel Monem Sanad

**Affiliations:** Construction and Building Engineering Department, College of Engineering and Technology , Arab Academy for Science, Technology and Maritime Transport (AASTMT), Elhorria, Heliopolis, Cairo 2033 Egypt

**Keywords:** Square steel silos, Finite element analysis, Lateral pressure distribution, Parametric study, Silo design guidelines, Engineering, Materials science

## Abstract

This study develops and validates a 3-D finite-element model for lateral pressures in square, flat-bottomed steel silos, challenging the applicability of conventional design methods. The model, using Mohr–Coulomb for wheat and surface-to-surface contact, closely matches observed pressures and demonstrates that slenderness h/a determines the pressure regime: Janssen-type asymptotic profiles in slender silos (h/a ≥ 7.5) change to Rankine-type linear profiles in squat silos (h/a ≤ 1.5). Therefore, using slender-silo formulae for squat designs may lead to inaccurate estimations of base pressures. A parametric study evaluates material influences: lateral pressure is significantly affected by Poisson’s ratio (raising from 0.28 to 0.45 more than doubles base pressure, + 110%) and wall friction µ, while it shows little sensitivity to Young’s modulus and cohesion. These results provide design-oriented recommendations for the safe and cost-effective sizing of silos across various geometries and granular materials.

## Introduction

### Background

**Fig. 1:**
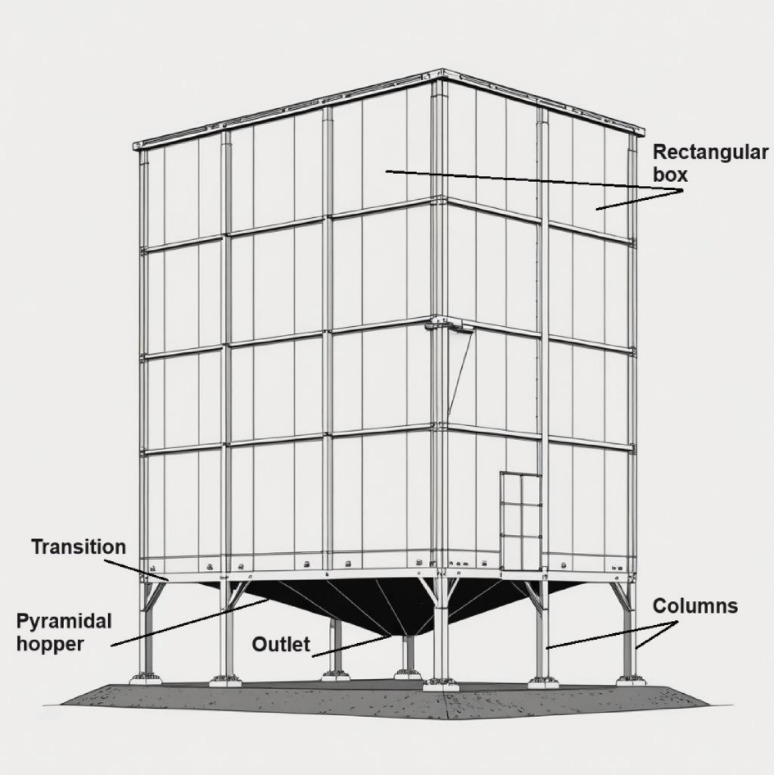
The main parts of a rectangular silo.

Silos are essential structures used throughout several industries for the storage of bulk materials, including grains, cement, and fertilizers^1–4^. Circular silos are often used because of their apparent simplicity in design and analysis. Rectangular silos provide substantial benefits, especially in scenarios where space efficiency is essential^5–8^. Rectangular silos (Fig. 1) are especially advantageous for storage facilities with spatial limitations since their design enables the optimum use of available space^9^. The construction process for rectangular silos often utilizes simpler methods, potentially leading to cost savings compared to the more intricate fabrication of curved surfaces in circular silos^5,7,10^.

The design of rectangular silos presents specific challenges owing to the complex nature of wall-filling pressure distribution^11^. Their implementation is complicated by a lack of developed design guidelines, resulting in failures such as wall buckling, corner cracking, and uneven discharge. Unlike circular silos, where the assumption of uniform pressure distribution is often employed, rectangular silos exhibit significant non-uniformity in pressure patterns, especially in flexible-walled structures. This fundamental phenomenon was later definitively confirmed by experimental measurements in square silos^10,12^, which clearly recorded the typical pressure gradient from the wall mid-span to the corner.

This non-uniformity results from the interaction between the inherent geometric asymmetry of rectangular silos and the wall deformability due to the weight of the stored granular material^5–7,9^. Understanding this complex pressure system is essential for maintaining the structural integrity and safety of rectangular silos.

The design of rectangular silos entails a complex interaction of structural mechanics, material science, and engineering principles^13,14^. The structural behavior of these silos is affected by many elements, such as the kind of stored material, loading conditions, and environmental conditions. Consequently, a comprehensive knowledge of the design, analysis, and applications of rectangular rigid wall silos is crucial for engineers and researchers in structural engineering^7^.

### Literature review

Over the past few decades, significant improvements have been made in the design and computational methods for granular material silos^15–18^. Several guidelines and recommendations have been provided for silo designers^5,6,9,19,20^. Silos have typically been designed based on uniform pressure distribution, as described by Janssen’s theory^21^ and numerous experiments have demonstrated this as a valid approximation for filling pressure^22^. Several standards and codes utilize the Janssen equation, which expresses wall pressure as indicated in Eq. 1.

1 $$\:{P}_{x}=\frac{A}{U}\cdot\:\frac{\omega\:}{\mu\:}\:(1-{\text{e}}^{-\frac{u}{A}\cdot\:\lambda\:\cdot\:\mu\:\cdot\:Z})$$

Where P_*x*_ represents the lateral wall pressure exerted on the wall. A represents the area, while U denotes the perimeter of the horizontal section. ω represents the specific weight of the material. µ represents the wall friction coefficient. λ represents the lateral pressure ratio. Z represents the depth measured from the free surface.

In circular silos, it is widely acknowledged that pressures are non-uniform at any given level, influenced by factors including eccentric filling, discharge, wall imperfections, and the arrangement of bulk material^23^. Recent updates to silo design regulations propose the use of local loads to account for bending moments resulting from non-uniform pressure distribution. Local unsymmetrical low pressures are generally more detrimental than extremely high symmetrical pressures^24^.

Nevertheless, the non-uniform pressures that develop around the perimeter of rectangular platform silos are of major relevance owing to the flexibility of the wall and the structure’s geometry^25^. The pressure distribution in circular silos is likewise influenced by the wall flexibility, as demonstrated by numerical models^7,26,27^. However, the impact is more pronounced in rectangular platform silos.

Many researchers, including^28–31^, have investigated the wall pressure of silos using the discrete element approach. Large silos are challenging to mimic using discrete element approaches due to their high calculation speed.

Sanad and others^16,18^ have contrasted the finite element approach with the discrete element method. Discrete element models provide qualitative estimates of flow patterns, while finite-element models offer quantitative predictions of pressure regimes. Researchers employed the finite element technique to assess the pressure applied to the walls of square silos^7^.

The mean wall-filling pressure could approximate Janssen’s estimations at any height, according to Goodey et al.^9^. However, filling pressures vary widely over horizontal sections. He also showed that the relative elastic stiffness of bulk solids and structural elements affects the distribution of wall-filling pressures and that slight changes in granular material characteristics can strongly affect silo structure design^5,7,32^.

The flexibility of silo walls transfers pressures from the center to the corners, according to Hilal et al.^7^. A rigid rectangular silo’s wall center has the highest pressure and the corners the lowest. The study examined how the silo slenderness ratio impacts rigid and flexible silo wall vertical load capacities.

Parameters like Young’s modulus, Poisson’s ratio, and internal angle of friction significantly affect design efficiency and wall pressure. Optimized silos provide superior durability, higher insulation, and increased resilience to environmental influences, resulting in reduced maintenance and energy use. The study emphasizes the importance of silo dimensions in influencing wall pressures, thereby warning against the generalization of small-scale model results to larger constructions.

### Research objectives

This study aims to accurately predict straining actions on rigid wall silos by developing a three-dimensional model of square granular material silos. This model will provide information regarding:

1. Load analysis on silo walls during filling: The investigation examined the forces that bulk solids apply to the walls of a square platform steel silo during the filling phase, providing observations into the lateral wall pressure distribution.
2. The study examined the influence of several mechanical properties on lateral wall pressure, including Young’s modulus, Poisson’s ratio, material density, grain-wall friction coefficient, cohesion coefficient, internal friction angle, and angle of repose.
3. The study examined the influence of silo dimensions and proportions on lateral wall pressure, highlighting the constraints of extrapolating pressure predictions from small-scale models to larger silos.
4. To examine the physical granular mechanisms that govern the observed pressures and obtain practical concepts for design.

These goals contribute to a better knowledge of silo design and operation, which may lead to enhanced structural integrity, efficiency, and sustainability in grain storage systems.

### Model limitations

The limitations of this study, as outlined in the paper, are fundamentally dependent on the conditions dictated by the silo’s structure and the interaction between the stored material and the silo walls. These constraints may be summarized as follows:

1. Wall rigidity: The silo walls are considered rigid, preventing the material in contact from moving perpendicular to the walls. This assumption may not exactly represent the behavior of actual steel silos, which might exhibit flexibility.
2. Flat bottom constraint: This restriction may not account for the implications of various bottom configurations or discharge systems.
3. The interaction between the stored material and the walls is entirely governed by friction. This simplification may not capture all the complex interactions present in real silos, such as adhesion or other surface effects.

These limitations are inherent to the model’s design and may affect the generalizability of the results to all types of silo configurations and operating conditions. However, they provide a necessary simplification to make the finite element analysis feasible while still offering valuable insights into the behavior of granular materials in steel silos.

## Finite element modeling

**Fig. 2:**
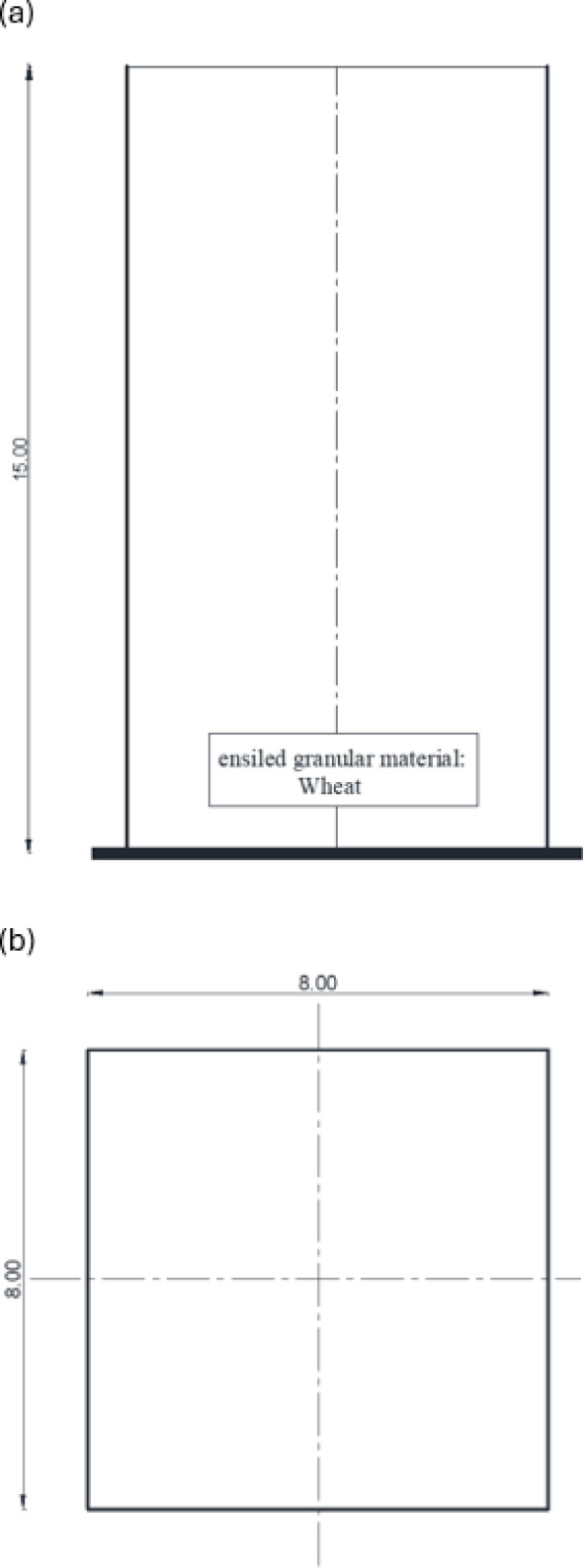
Geometry of the silo model. (a) Elevation perspective of the model; (b) Plan perspective of the model. (All dimensions in meters).

A 3D finite element model (FEM) is developed utilizing ABAQUS^33^ software to analyze the stresses on the walls of a square silo. Figure 2 demonstrates the geometry of the finite element model that was used in the numerical analysis. The 8-node hexahedral elements (C3D8R) are used to discretize the silo structure and the stored material. This element formulation was chosen for its computational efficiency in simulating extensive contact issues and its capacity to reduce shear locking in bending-dominated situations^34^. A possible limitation of reduced-integration elements is the occurrence of zero-energy modes (hourglassing); however, this was effectively managed through the element’s integrated hourglass stabilization algorithm, and the hourglass energy was monitored during all analyses to ensure it remained insignificant compared to the total strain energy^35^. The element’s durability in managing significant deformations and intricate contact interactions renders it especially appropriate for geotechnical applications concerning granular materials^33^. Every element has three translational degrees of freedom. The generated finite element model was employed to evaluate the parameters influencing the loads exerted on the silo wall. The finite element findings have been examined and validated against another finite element model developed by Sanad^23^ and the conventional and extensively used method in silo guidelines, the Janssen equation^21^.

### Description of the finite element model

**Fig. 3:**
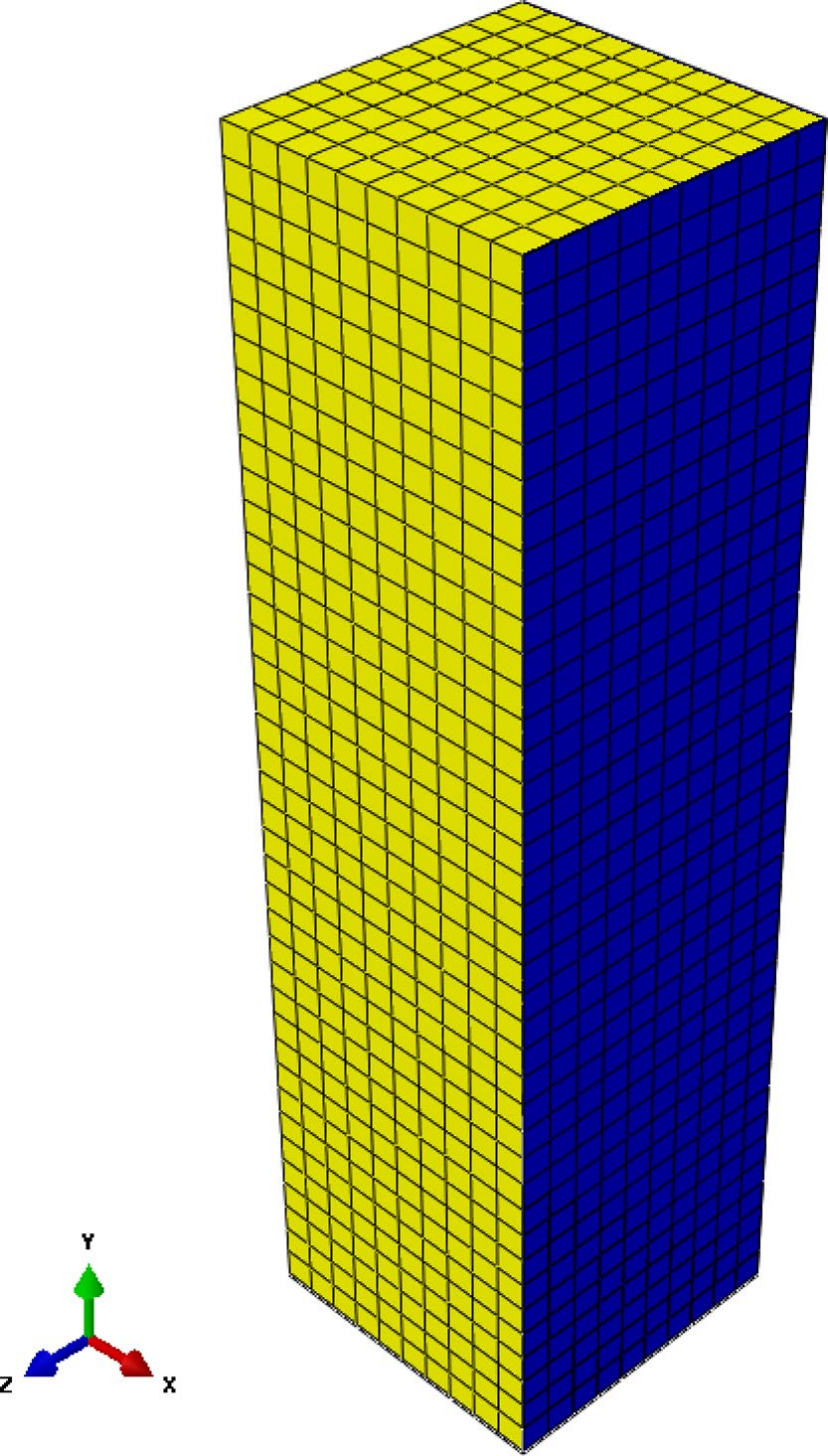
Finite element model (ABAQUS model).

Granular material having a density of 800 kg/m³ is stored in a square silo of 15.0 m in height and 8.0 m in width. The incremental filling process was simulated to precisely capture the development of stresses inside the granular material. The wheat volume was divided into 15 horizontal layers, each reaching 1.0 m in height. The model change function in ABAQUS was used to successively activate these layers throughout a series of geostatic analysis phases, starting with the bottom layer. This strategy guarantees a stable numerical solution by avoiding the significant initial distortions that would arise from the immediate application of the whole load. Moreover, it accurately simulates the development process, facilitating the path-dependent change in stresses and wall pressures during filling^36^. The silo wall was deemed a stiff structure that inhibits lateral movement along the height of the silo. The finite element model was created with data obtained from Sanad’s model. Figure 3 demonstrates that only one-quarter of the silo can be seen due to symmetry. The model has three components: the granular materials, the silo walls, and the base. The finite element findings were compared with the numerical model of Sanad at the mid-span of the silo along its height. We confirmed mesh independence by using three systematically refined C3D8R meshes, with the global size reduced by half at each iteration and local refinement applied at the wall–base junction. Convergence was considered attained when the maximum base lateral pressure and the mid-height average wall pressure profile varied by less than 2% between consecutive revisions.

### Bulk solids

The granular material that is stored is modeled as isotropic, which implies that its properties are consistent in all directions. The granular materials are simulated using an elasto-plastic model, which has both elastic and plastic characteristics. Specifically, the Mohr-Coulomb yield criterion is employed to determine the plastic behavior, which is well-suited for granular materials such as wheat. The yield behavior of the material under a variety of stress circumstances is accurately represented by this criterion, which takes into account parameters such as internal friction angle and cohesiveness. The Mohr-Coulomb (M-C) elastoplastic model was used since it is the accepted standard for modeling shear failure and pressure-dependent behavior in granular materials^37^. The formulation directly employs basic strength values derived from regular laboratory measurements.

In this model, the internal friction angle (φ) determines the gradient of the linear yield surface in the principal stress space, controlling the material’s shear strength and the critical stress ratio at which plastic yielding begins. The cohesion (c) denotes the shear strength at zero normal stress; a value of c = 0 kPa was deliberately selected to illustrate the entirely non-cohesive, frictional characteristics of dry wheat grains, a decision corroborated by our parametric analysis. The angle of repose (φ_r_), while a valuable empirical indicator of a material’s static stability, is not a direct input parameter for the M-C constitutive model. This was used for preliminary model validation to confirm that the simulated material’s shear response corresponded with the established physical properties of wheat.

In contrast, the steel silo structure is analyzed using a more straightforward linear elastic model, which presupposes a direct relationship between stress and strain. The mechanical and physical characteristics of wheat and steel silo walls were obtained from Sanad’s model. The mechanical and physical properties of wheat and steel are shown in Tables 1 and 2, respectively.

**Table 1:** Mechanical properties for stored granular material (Wheat).

No.	Property	Value
1	Young’s modulus, E (MPa)	20
2	Poisson’s ratio, ν	0.35
3	Density, ρ (kg/m^³^)	800
4	Grain-wall friction coefficient, µ	0.33
5	Cohesion, c (kPa)	0
6	Internal friction angle, φ (degrees)	28
7	Angle of repose, φ_r_ (degrees)	25.48

**Table 2:** Mechanical properties for Steel.

No.	Property	Value
1	Young’s modulus, E (GPa)	210
2	Poisson’s ratio, ν	0.30
3	Density, ρ (kg/m^³^)	7500

### Granular material−wall interaction

A surface-to-surface contact model is employed to simulate the interaction between the silo wall and the granular material (wheat). The interaction between the stored granular material and the silo wall is simulated using a contact pair. This interaction occurs between the inner surface of the silo wall and the outer surface of the granular material. The coefficient of friction that exists between bulk solids and the silo wall is assumed to be uniform throughout the height of the silo, as shown in Table 1. This model utilizes the Coulomb friction model to describe frictional interactions during sliding.

### Boundary conditions

**Fig. 4:**
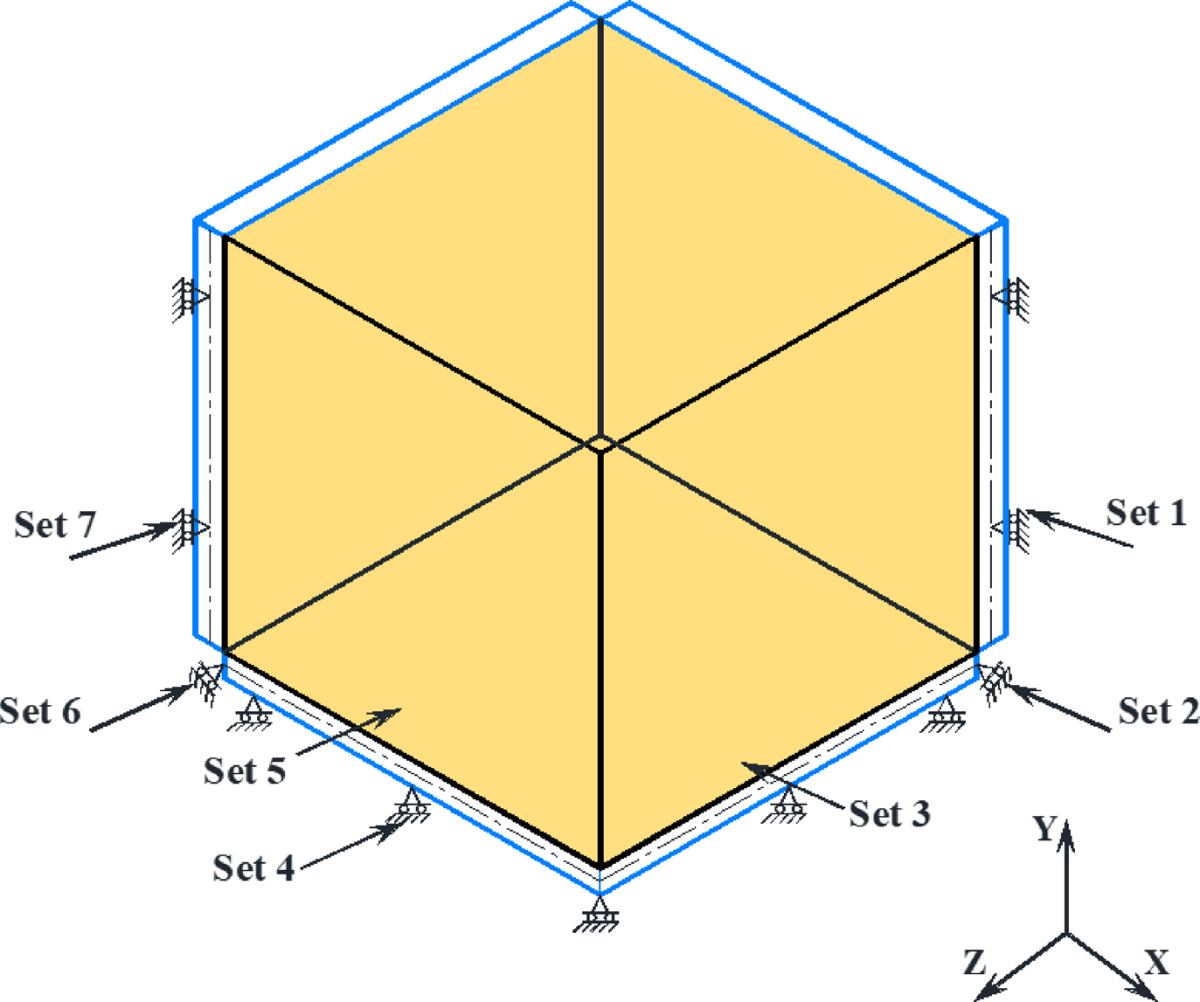
The assignment of boundary conditions.

All finite element models investigated so far had a flat base, and the structure’s bottom has been vertically constrained in the Y-direction, as seen in Fig. 4. The assignment of boundary conditions for the granular materials and silo walls was established only along the axes of symmetry in the x and z dimensions to prevent normal displacements regarding the symmetry plane. The assignment of boundary conditions for the quarter silo were defined in detail as follows:

Set 1: Prevent the lateral movement of the wall along the X and Z axes.

Set 2: Prevent the lateral movement of the base along the X-axis.

Set 3: Prevent the lateral movement of the granular materials in XSYMM.

Set 4: Prevent the movement of the base in the vertical direction along the Y-axis.

Set 5: Prevent the lateral movement of the granular materials in ZSYMM.

Set 6: Prevent the lateral movement of the base along the Z-axis.

Set 7: Prevent the lateral movement of the wall along the X and Z axes.

## Results and validation

**Fig. 5:**
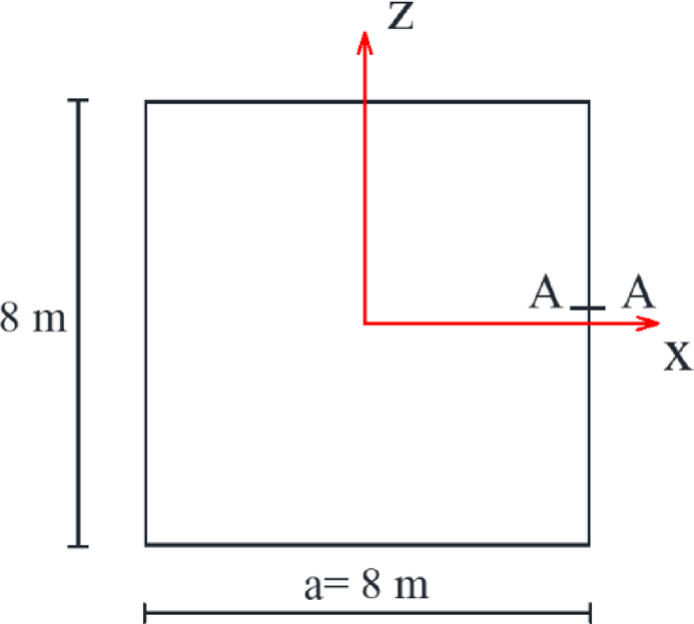
Plan view of the section cut.

The results of the finite element are contrasted and validated against the analytical results of the Janssen equation^21^ as well as the previous numerical model results developed by Sanad^23^. The results obtained from the Janssen equation and Sanad model are only available at the mid-span of the silo wall throughout its height, as seen in Fig. 5. The bottom of the silo is the location of the zero point on the vertical axis.

### Comparison with the Janssen equation

The Janssen equation is one of the original and most commonly employed approaches for silo design. It established the basis for formulating the differential equation that controls the equilibrium of granular materials in a silo. The model, originally designed for circular silos with axisymmetric layers, assumes a constant ratio between horizontal and vertical pressures. Modern silo design guidelines mostly use this approach to determine the pressure applied to silo walls. The method has been extended to cover non-circular shapes, such as square silos, by equating them to circular counterparts with an equivalent hydraulic radius (R)^38^.

**Fig. 6:**
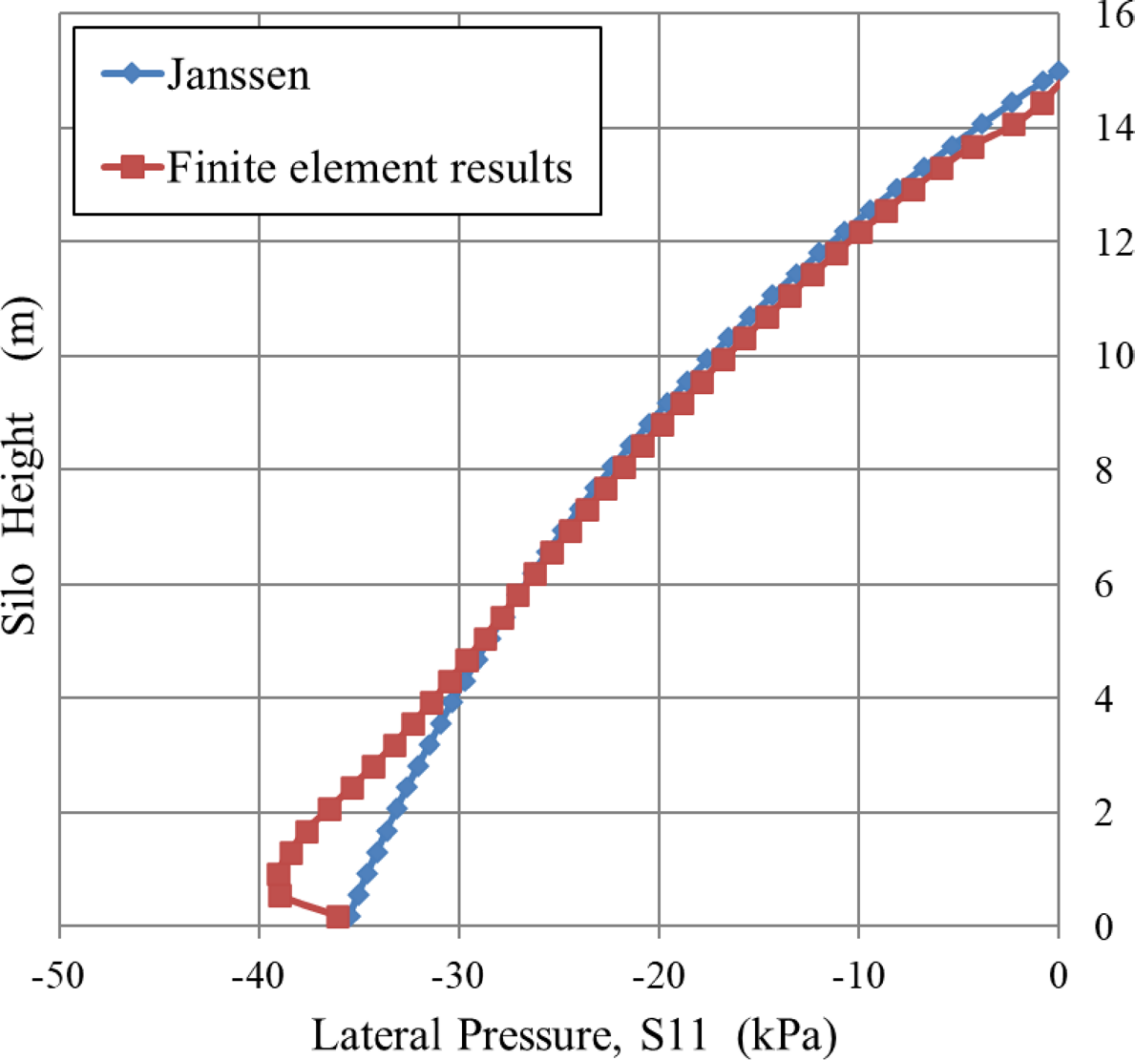
Comparison of lateral pressure between finite element results and Janssen predictions along the height of the silo.

The comparison was conducted between the results derived from finite element analysis and those predicted by the Janssen equation for a rigid-walled silo of 8× 8 × 15 m, loaded with wheat, as seen in Fig. 6.

Figure 6 illustrates a comparison between the finite element analysis results and Janssen’s predictions at the wall’s mid-span. The finite element findings roughly align with Janssen’s predictions for the center and top parts of the silo wall. In the bottom part, the finite element values surpass Janssen’s predictions due to a pronounced end effect at the interface between the granular materials and the silo wall.

The phenomenon results from a discontinuity in the boundary conditions at the base of the silo. The rigid, level surface restricts the vertical displacement and shear deformation of the granular material, limiting the complete activation of wall friction in this area. The obstacle to shear stress creation prevents the development of the comprehensive stress-redistributing arching mechanism that is essential to Janssen’s idea. As a result, the vertical stress on the floor continues at an increased level, resulting in an associated increase in lateral pressure on the wall at the base (*S11* = *K*⋅*σ*_*v*​_).

The complex interaction at geometry discontinuities is a noted constraint of the Janssen equation, which presumes a completely flexible vertical membrane and neglects end effects at rigid boundaries. The finite element model’s ability to accurately represent this particular pressure rise is a significant benefit, since this area is often essential to structural design. Similar end effects have been shown by other researchers^5,7,19^ and are specifically outlined and measured in experimental experiments on square silos conducted by Goodey et al.^39^.

### Comparison with Sanad’s numerical model

**Fig. 7:**
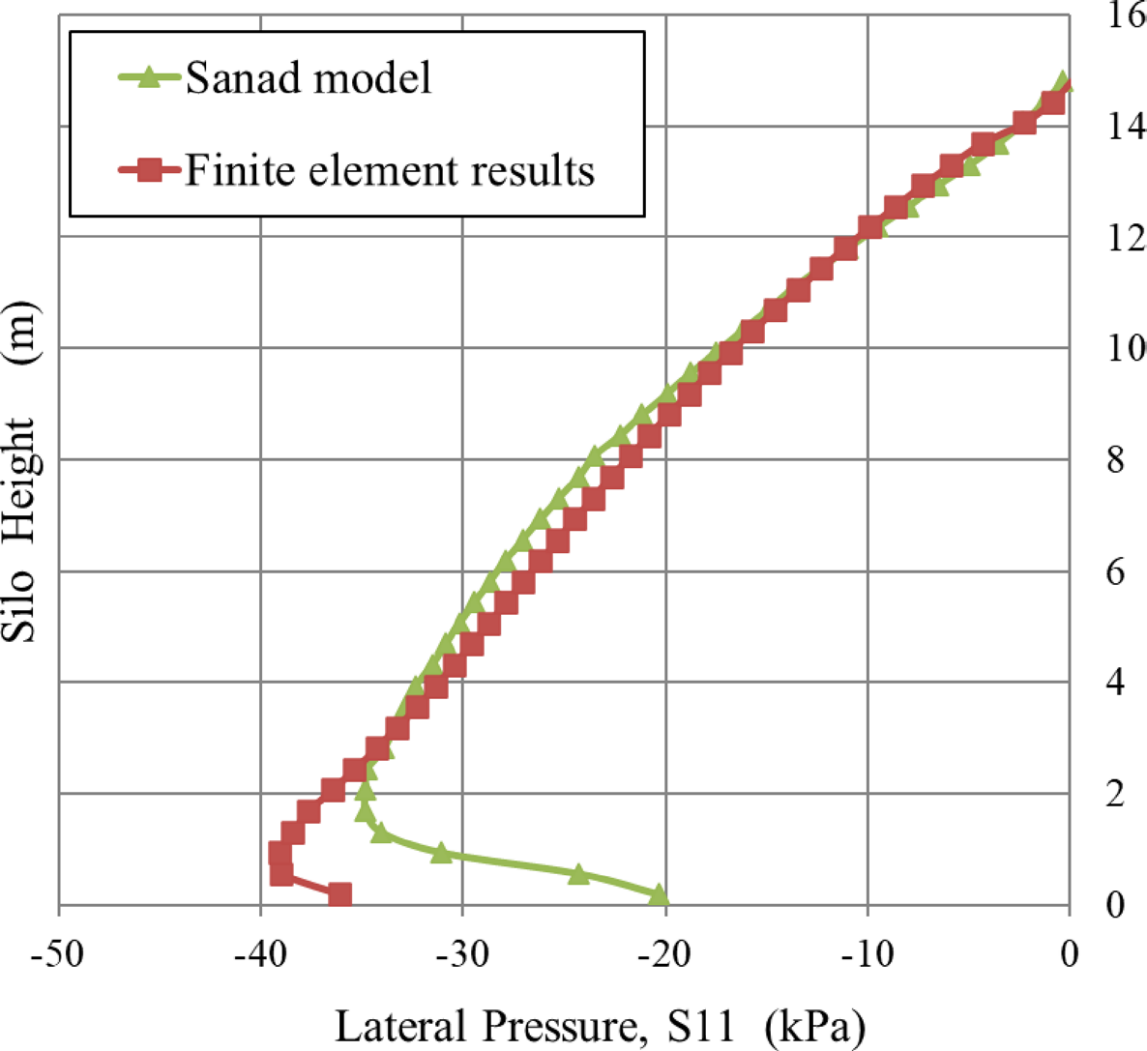
Comparison of lateral pressure distribution along the silo height between the finite element analysis and the results reported by Sanad.

The static wall pressure values derived from the ABAQUS finite element model align closely with those predicted by Sanad’s numerical model, as shown in Fig. 7. The divergence between the two finite element methods can be attributed to variations in the anticipated material properties. The first model, created in FORTRAN^40^, used a non-linear elastic material formulation based on the Boyce constitutive law^41^, which more precisely represents the complex stress-strain relationship observed in real materials. In contrast, the ABAQUS model employs a linear elastic material model according to Hooke’s law^42^, where stress is directly proportional to strain. The fundamental discrepancy in constitutive modeling accounts for the variations in their relative results.

The finite element model was evaluated by comparing its results with two independent approaches: one using the Janssen equation and the other derived from Sanad’s numerical model. The comparative findings demonstrated reasonable agreement, indicating that the finite element model precisely represents the lateral pressure behavior throughout the height of the silo. This close alignment validates the capability of the model to simulate the structural response under given conditions. The alignment between the methods confirms the credibility of the finite element model, indicating its reliable use for further analyses and design tasks in similar loading situations.

## Influence of material properties

In order to examine the impact of a variety of mechanical parameters on the lateral wall pressure of the silo, a comprehensive parametric study was conducted. The study included the modification of specific parameters while maintaining the remainder of the parameters constant, resulting in the following results:

### Young’s modulus, E

**Fig. 8:**
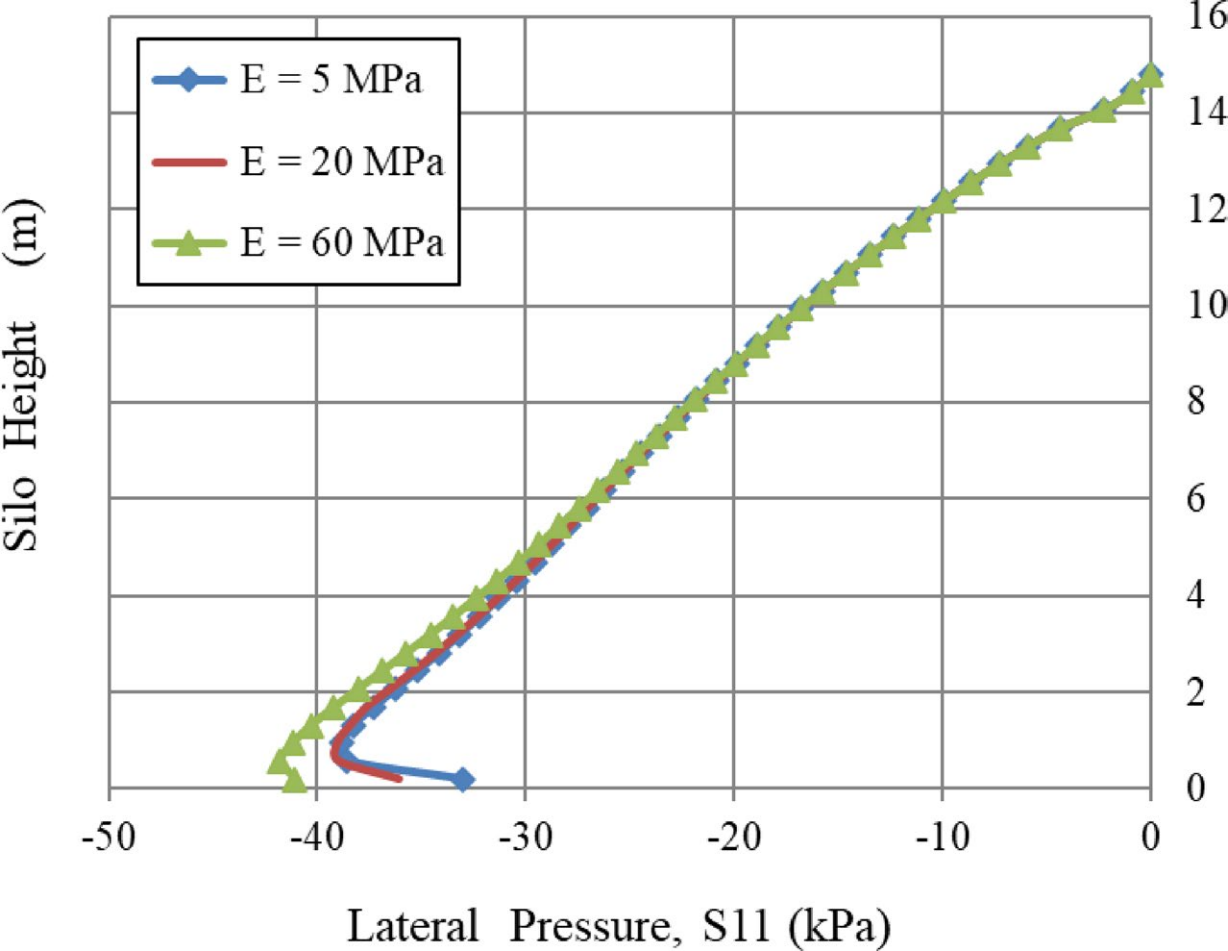
Influence of Young’s modulus, E.

The curves are closely aligned, indicating that an increase in the modulus of elasticity (E) leads to a little change in the lateral wall pressure distribution throughout the height of the silo, as illustrated in Fig. 8. At lower heights, the pressure differences among the three curves are more pronounced, with stiffer materials (higher E) often exhibiting slightly lower wall pressures. However, its impact was negligible in the upper and middle sections of the silo (Fig. 3).

From 5 to 60 MPa, E increased by 1100%. The lateral pressure S11 at the bottom increased from 33 to 41 kPa, indicating a 24.2% rise. While remaining unchanged for the top and middle sections. This indicates that the variation in lateral pressure resulting from a change in Young’s modulus is significantly influenced by the material’s deformation state [small deformations (elastic region)/large deformations (failure)].

### Poisson’s ratio, ν, and density, ρ

**Fig. 9:**
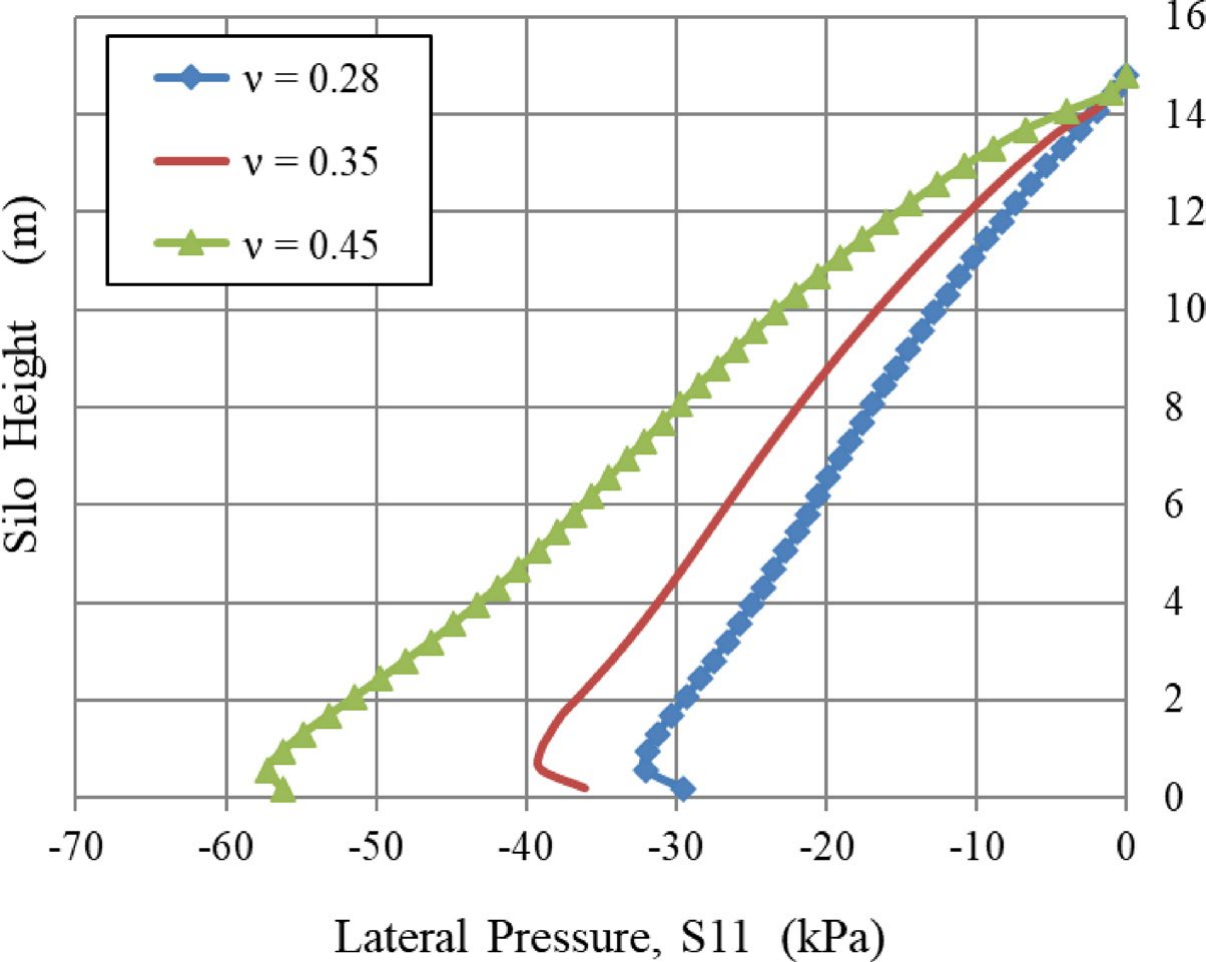
Influence of Poisson’s ratio, ν.

**Fig. 10:**
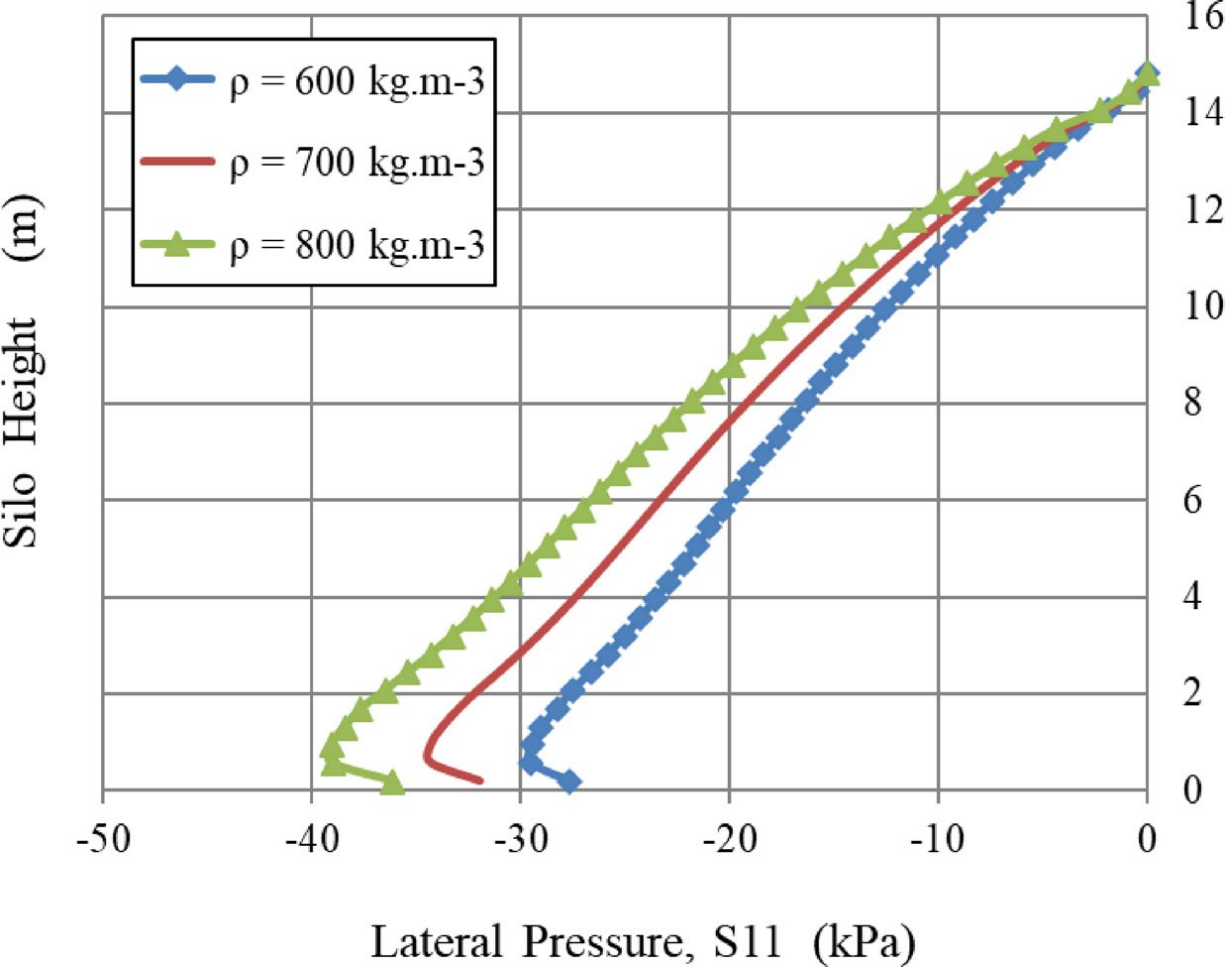
Influence of density, ρ.

The lateral wall pressure was positively correlated with Poisson’s ratio (v) and material density (ρ), as illustrated in Figs. 9 and 10, respectively.

As Poisson’s ratio (v) increases, the lateral pressure exerted on the silo walls increases. The analysis demonstrates a significant characteristic of material behavior: lateral pressure shows a very sensitive and non-linear relationship with Poisson’s ratio. A little rise in v results in a disproportionately greater increase in lateral pressure, as seen by the computed variations. A 25% increase in ν (from 0.28 to 0.35) results in a 38% rise in lateral pressure; a subsequent 29% increase in ν (from 0.35 to 0.45) leads to an additional 52% increase, and the total 61% rise in ν (from 0.28 to 0.45) more than doubles the lateral pressure, resulting in a 110% overall escalation. The significant amplification effect is determined by the formula K₀ = ν/(1 - ν), in which a rise in ν reduces the denominator, hence substantially enhancing its influence on the resultant stress state. K_o_ denotes the coefficient of lateral earth pressure at rest.

The graph analysis indicates a straight linear relationship between material density (ρ) and the resulting lateral pressure (S11). The proportionality is dictated by the basic concept of geostatic stress, whereby the vertical pressure (σ_v_) is determined by the equation ρ * g * h, and the lateral pressure (S11) is a function of that vertical pressure, generally expressed as S11 = K₀*ρ*g*h. (where g is the acceleration due to gravity and h is the height of the bulk solids). Thus, a certain percentage increase in density leads to a corresponding percentage increase in lateral pressure at any particular depth. For instance, increasing the density by 16.7% (from 600 kg.m⁻³ to 700 kg.m⁻³) yields an associated 16.7% escalation in S11; likewise, a 33.3% increase in density (from 600 kg.m⁻³ to 800 kg.m⁻³) leads to a 33.3% augmentation in lateral pressure. This direct proportionality emphasizes that density is a fundamental and reliable determinant of actual lateral stress in geotechnical systems.

In Figs. 9 and 10, all curves converge near the apex of the silo, indicating that Poisson’s ratio and material density have little influence at these heights.

### Grain-wall frictional coefficient, *µ*

**Fig. 11:**
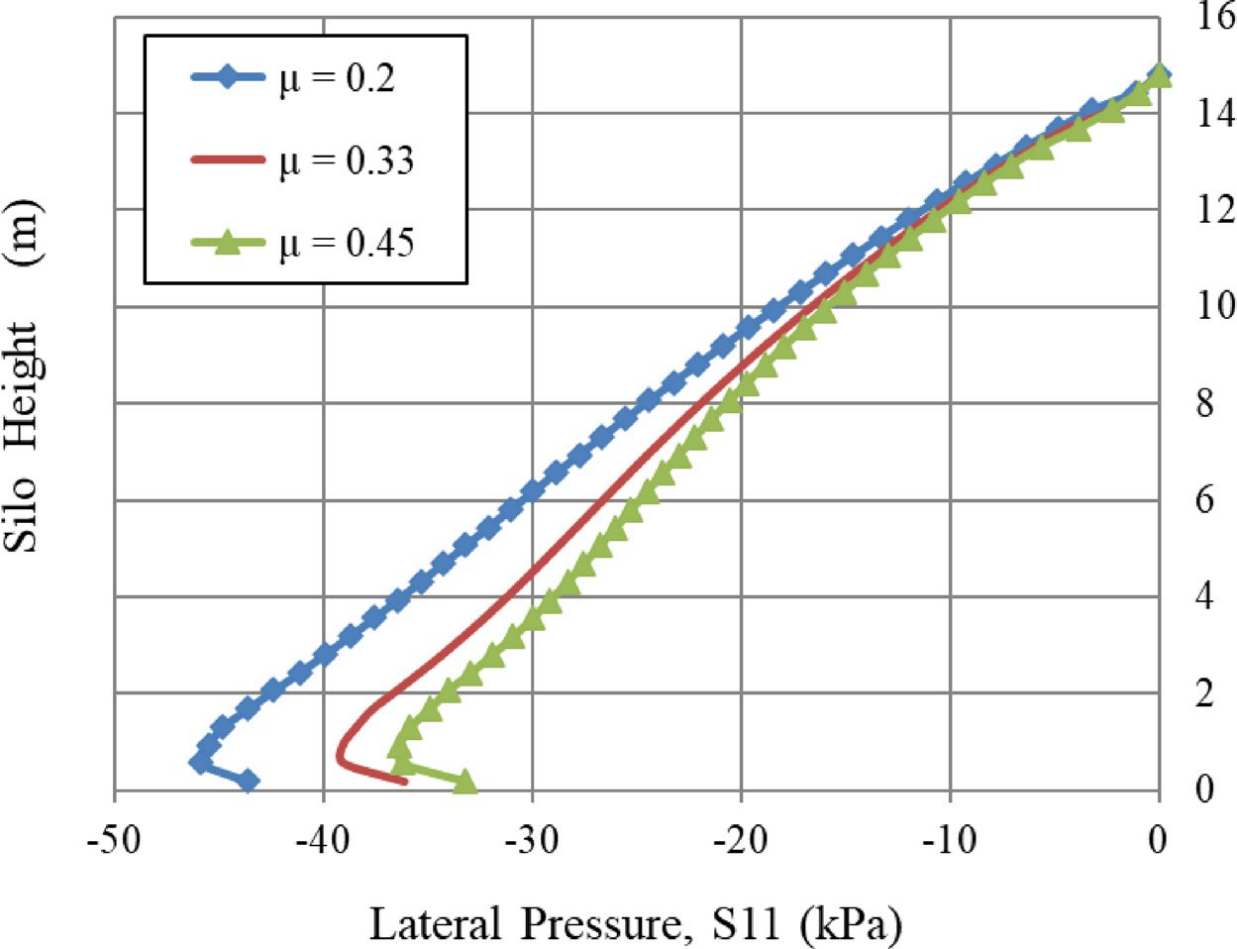
Influence of grain-wall Frictional coefficient, µ.

The analysis shows a fundamental inverse relationship between the grain-wall frictional coefficient (µ) and the corresponding lateral pressure (S11), as seen in Fig. 11. This occurs due to an elevated interface friction coefficient, which allows greater shear stress at the contact between the bulk solid and the wall. This improved shear transfer promotes arching effects, whereby a larger fraction of the soil’s weight is redirected vertically through the soil mass, rather than being transferred horizontally to the wall. Thus, a rise in µ results in a significant drop in S11. A 65% rise in µ (from 0.2 to 0.33) may lead to a significant reduction in lateral pressure, potentially between 30 and 40%, whilst a 125% increase (from 0.2 to 0.45) might reduce S11 by over 50%. This highlights that the grain-wall friction coefficient is an essential design parameter for minimizing lateral pressures on earth-retaining structures. All curves merge at the top of the silo, indicating that frictional effects are more pronounced at the base level.

### Cohesion, c and repose angle, φ_r_, and angle of internal friction (φ)

**Fig. 12:**
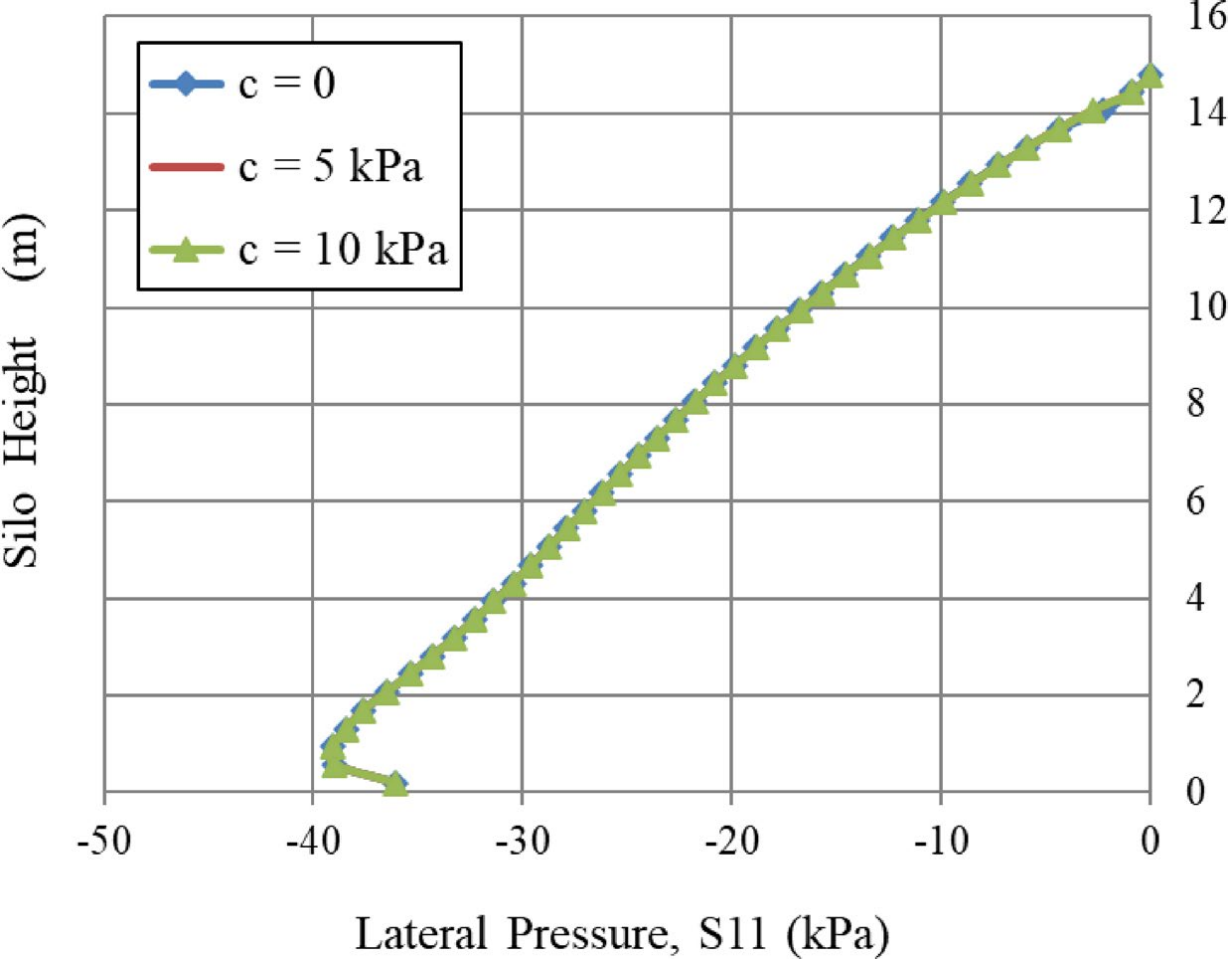
Influence of cohesion, *c*.

**Fig. 13:**
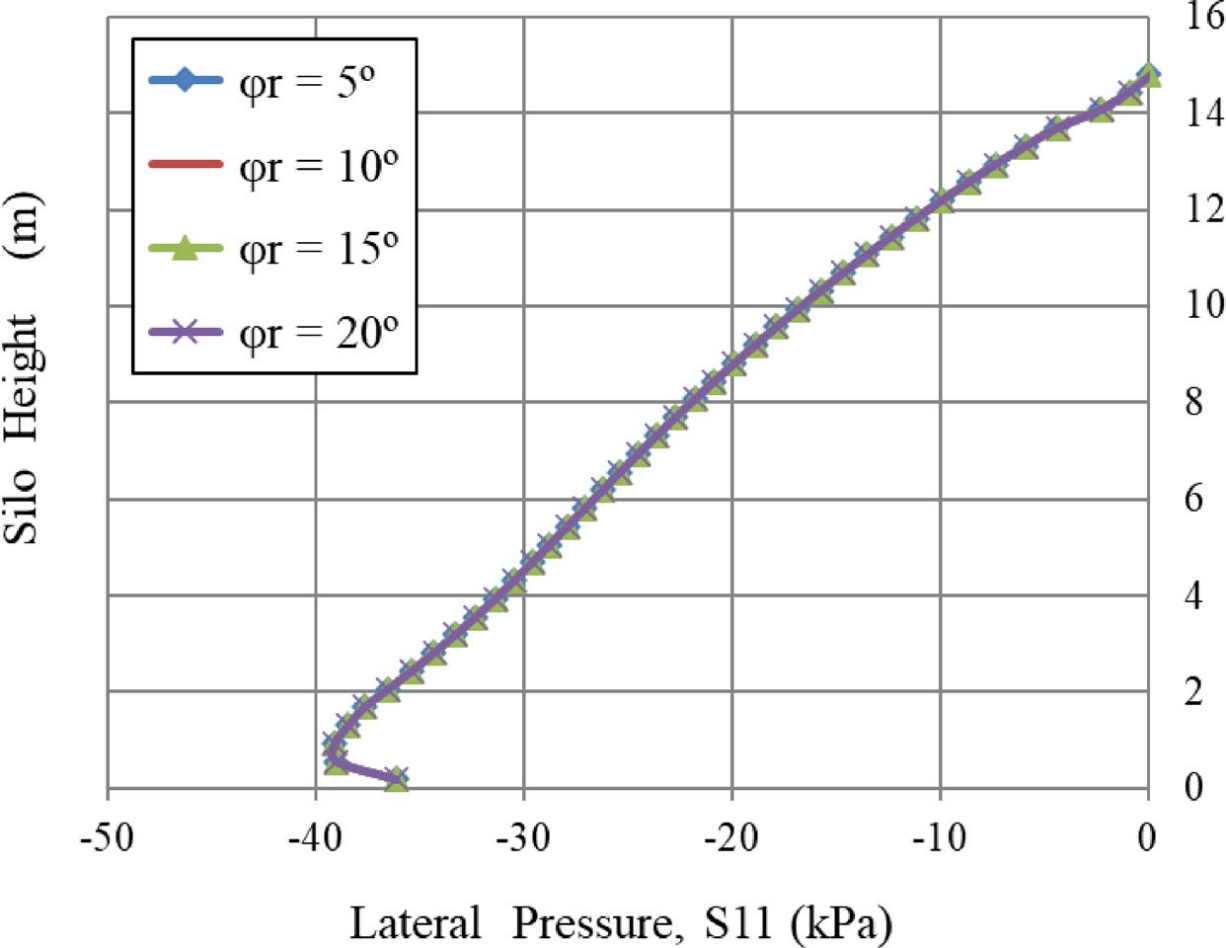
Influence of repose angle, φ_r_.

In Figs. 12 and 13, the three curves are completely overlapped. This indicates that cohesion (c) and the repose angle do not affect the lateral pressure distribution in this context. This indicates that the bulk solid behaves as a cohesionless solid or that the influence of cohesion is negligible under this stress regime. Despite cohesion (c) values reaching 10 kPa, lateral pressure remains constant. These findings are restricted to the FEM framework and the static filling situations considered; alternative methods, such as DEM, may exhibit different sensitivity to cohesion under varying flow or loading conditions.

The angle of repose (φ_r_) is not a direct variable in analytical silo pressure models, notably Janssen’s^21^. According to Rotter^24^, φ_r_ provides as an empirical measure for defining a material’s shear deformation under gravitational forces, however, it does not affect the stress distribution during static filling. Lateral pressure is mostly dependent upon the angle of internal friction (φ) and the wall-friction coefficient (µ). This concept is validated by numerical studies; for example, Vidal et al.^43^ performed a parametric finite element study revealing that variations in φ_r_ between 5° and 30° led to a negligible alteration of under 2% in lateral pressure for steel silos.

**Fig. 14:**
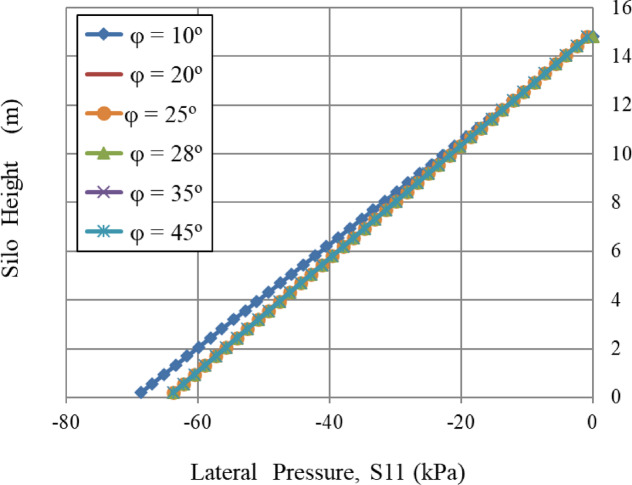
Influence of internal angle friction, φ.

Figure 14 reveals a crucial understanding of silo pressure: in order to isolate the effect of the angle of internal friction, both the angle of repose (φr) and the grain-wall friction coefficient (µ) were assigned a value of zero for this investigation. Under these controlled settings, results indicate that the practical effect of the internal friction angle (φ) is marked by substantially lower returns. A significant 350% increase in φ (from 10° to 45°) results in a slight 7.3% decrease in lateral pressure, from 68.7 kPa to 63.8 kPa. This signifies that the primary internal arching mechanism, only influenced by φ in the model, attains remarkable efficiency at a minimal limit. The majority of its stress-reducing impact is attained at a low φ value, making further increases in the internal friction angle almost insignificant for lateral load calculations. Consequently, in this particular circumstance, the lateral pressure stays almost constant for any internal friction angle over a particular limit value.

The parametric study revealed significant variations in lateral wall pressure dependent on the mechanical properties of the stored granular material and the silo structure. The silo’s base was notably influenced by the following mechanical properties, in order of their impact: (1) Poisson’s ratio (ν), indicating the material’s ability to expand laterally under vertical compression presented a robust positive correlation with wall pressure, due to the non-linear relationship of the lateral earth pressure coefficient, K₀ = ν/(1 - ν); (2) The grain-wall frictional coefficient (µ) demonstrated an inverse relationship with pressure, resulting from the increased shear transfer and arching mechanism; and (3) Young’s modulus (E), a measure of material stiffness, demonstrated a more diminished but apparent effect, especially in the lower section of the silo where deformation is most constrained.

Similarly, the internal angle of friction, an essential factor in granular mechanics, significantly influenced wall pressures until a certain limit was reached. In contrast, the lateral wall pressures were slightly influenced by the dilatation angle and the cohesiveness of the granular material, which relates to volume variations during shear. Figures 8, 9, 10, 11, 12, 13 and 14 illustrate these findings, which provide valuable insights for enhancing silo design and understanding the intricate relationship between structural loads and material properties.

## Influence of silo dimensions

### Effect of planform dimensions

**Fig. 15:**
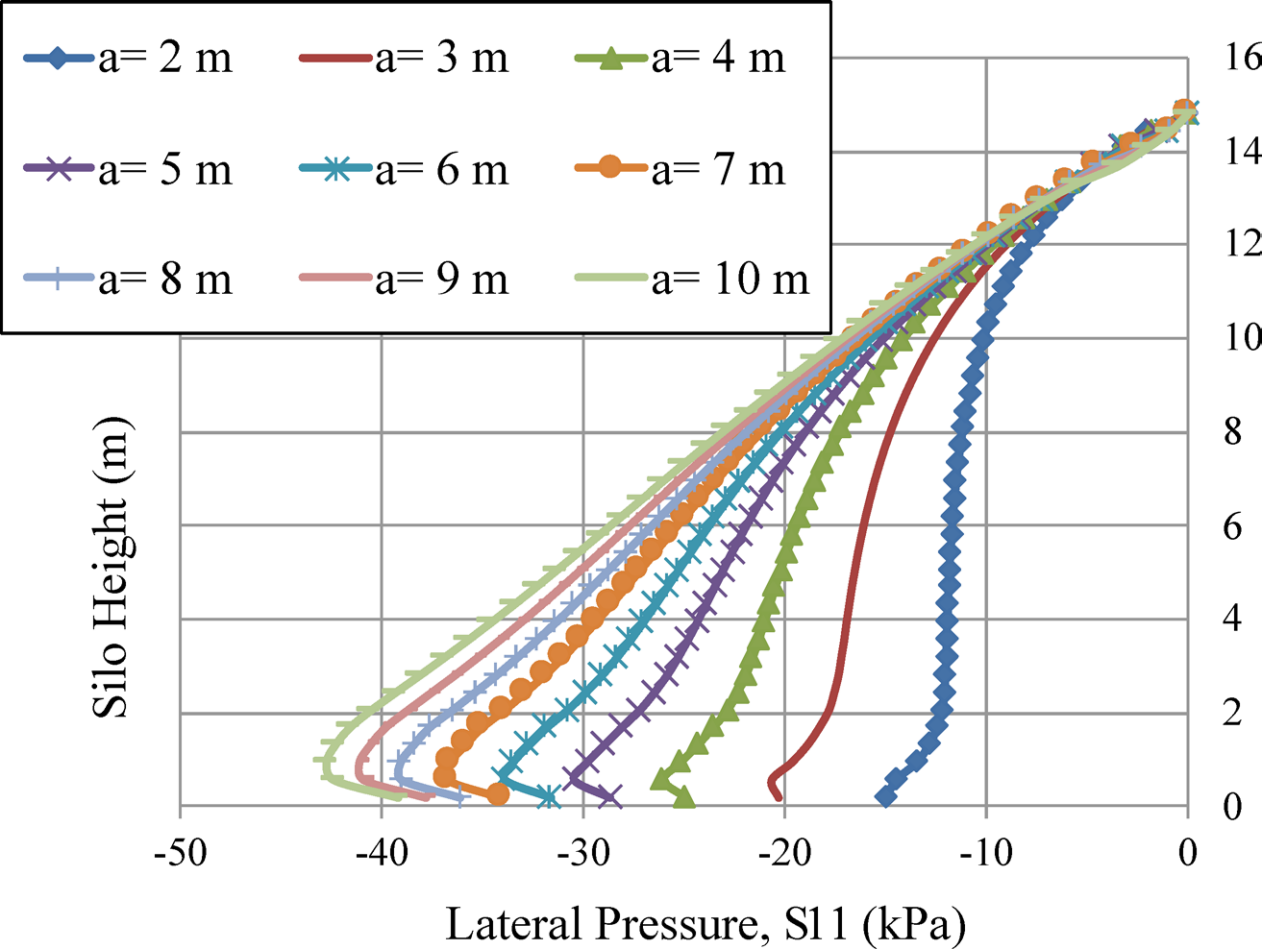
Lateral wall pressure of different planform dimension models.

**Fig. 16:**
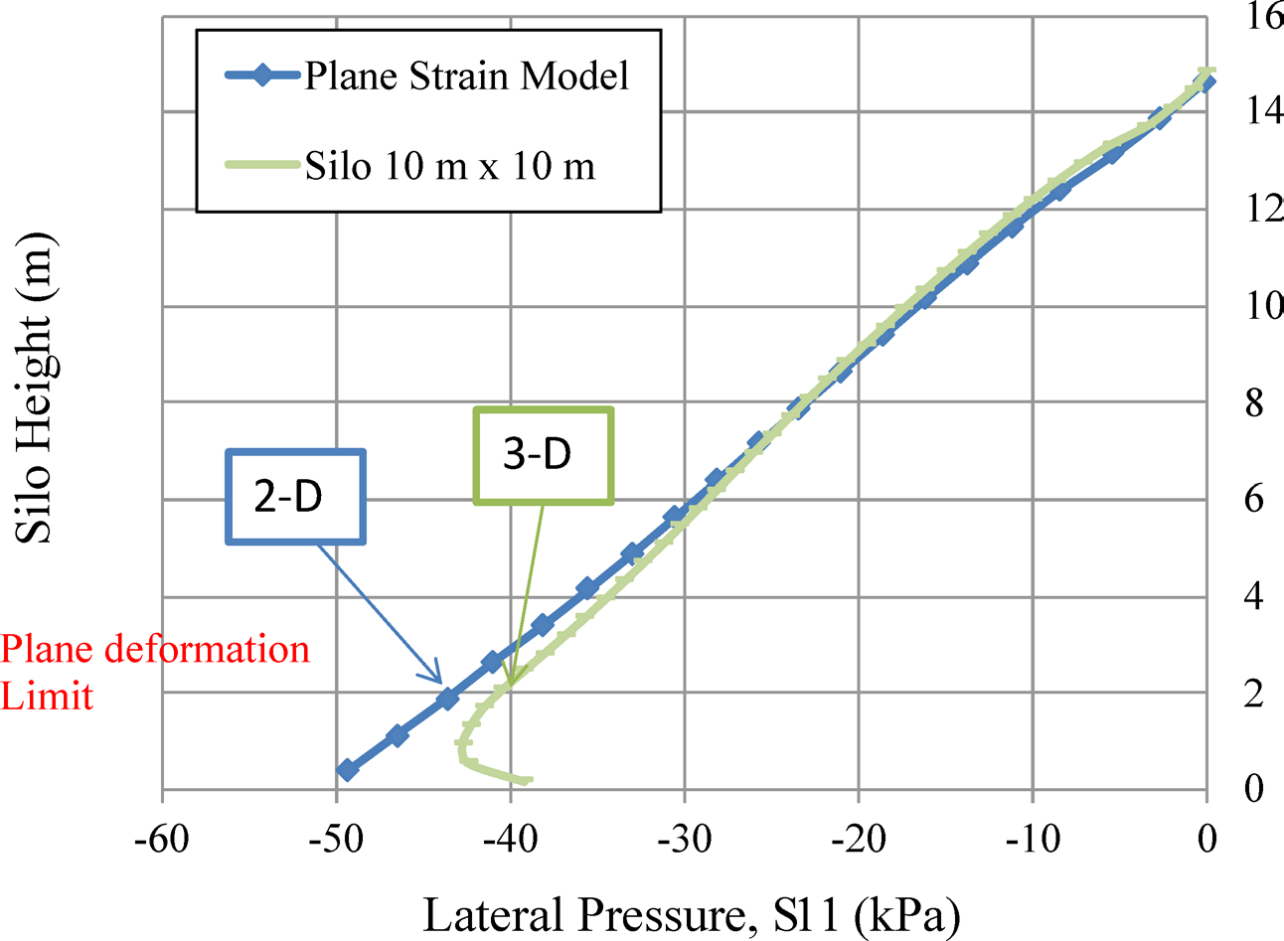
Lateral wall pressure limit of plane deformation (plane strain).

In order to examine the impact of silo planform dimensions on lateral wall pressure, several square models were developed with different planform dimensions (a) with the same height (h). All other factors remain unchanged. The wall pressures of all square models were compared. Figure 15 illustrates that wall pressure increases with the dimension of the silo planform. The pressure is asymptotic for slender silos (a = 2, h = 15, h/a = 7.5^38^, hence confirming Janssen’s equation. The pressure tends to be linear for squat silos (a = 10, h = 15, h/a = 1.5^38^ with the plane deformation results as the limit, as shown in Figs. 15 and 16. Otherwise, the pressure tends to be P_x_=k_a_.γ.h. Where k_a_ represents the ratio of horizontal pressure to vertical pressure, and γ is the weight per unit volume of the bulk solids.

Figure 15 is a credible curve and confirms Janssen’s equation applicability for slender silos, and the plane deformation limit for squat silos. Therefore, it is not appropriate to predict the wall pressure of a squat silo using methods that are based on the computation of slender silos^7^.

### Effect of silo proportions

**Fig. 17:**
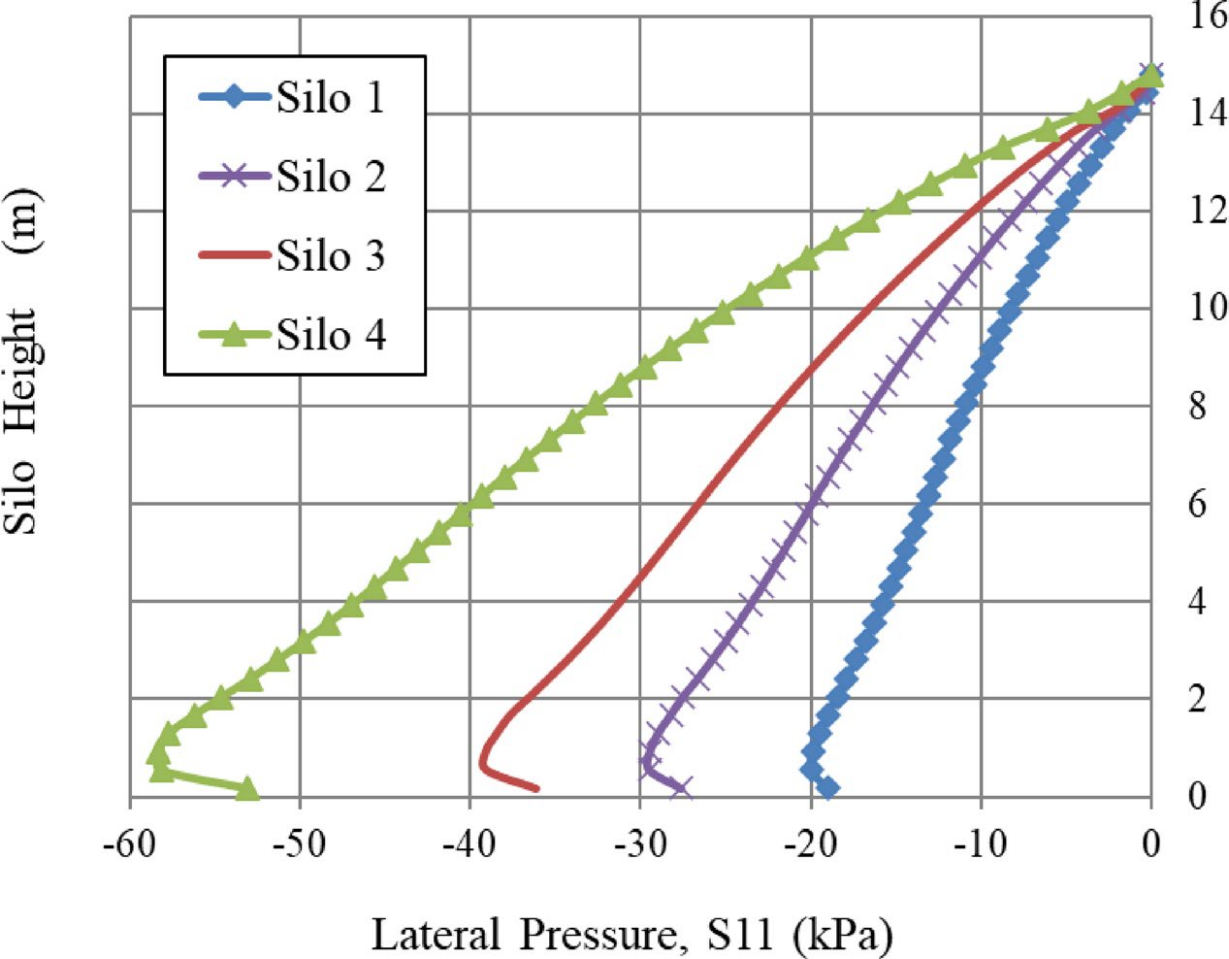
Different lateral pressures for different square silo dimensions.

To investigate the lateral wall pressures on these silos, several square models have been developed with different scale ratios. Additionally, to validate the lateral pressure ratios at the base of the silo concerning the maximum lateral pressure and the vertical pressure. The lateral pressure lines have identical shapes, while their values vary. The pressure ratios stay almost constant, exhibiting very slight changes, as seen in Fig. 17; Table 3.

Table 3 illustrates that increasing the silo’s scale results in higher absolute lateral and vertical pressures due to the increased weight of the stored material; yet, the key dimensionless pressure ratios remain fairly constant across different scales. For example, when the dimensions of the silo are tripled (scale ratio 3), the bottom lateral pressure remains around 92–95% of the maximum lateral pressure on the wall, while the lateral wall pressure is about 58–61% of the vertical pressure. This consistency indicates geometric similarity and scale independence in the silo’s behavior: the pressure distribution profile maintains a fundamentally the same form regardless of the silo’s absolute dimensions. In other terms, consistent geometric scaling does not modify the proportionate pressure distribution. The scale-invariant pressure ratios highlight the reliability of the FEM model—parametric simulations conducted on a smaller silo accurately represent the normalized pressure distribution in a larger silo. This verifies that pressure distribution patterns are mostly maintained despite uniform scaling, facilitating reliable extrapolation of the FEM findings for the development of full-size square silos.

**Table 3:** Silos with different dimension ratios.

Silo no.	x-sec	Height	Scale Ratio	Lateral Pressure (kPa)		Vertical Pressure (kPa)	Lateral to Max. Lateral Pressure Ratio	Lateral to Vertical Pressure Ratio
				At the bottom level	Maximum			
1	4 × 4	7.5	1	−19.012	−19.929	−32.724	0.95	0.58
2	6 × 6	11.25	1.5	−27.602	−29.469	−46.565	0.94	0.59
3	8 × 8	15	2	−36.119	−39.011	−60.071	0.93	0.60
4	12 × 12	22.5	3	−53.053	−58.147	−86.76	0.92	0.61

## Discussion

The results of this analysis provide substantial insights into the intricate relationship among material characteristics, silo geometry, and lateral wall pressures in rigid steel silos. The assessment of the finite element model against Janssen’s equation and Sanad’s numerical simulations confirms its reliability in predicting pressure distributions inside square silos. The tight alignment in the mid-to-upper parts of the silo wall validates the utility of traditional concepts for these regions. The discrepancy seen at the base, where finite element projections surpass Janssen’s values, highlights the shortcomings of conventional analytical approaches in accurately representing localized end effects. This phenomenon, along with previous studies by Goodey et al.^5^ and Hilal et al.^7^, highlights the need for advanced computational methods for addressing stress concentrations at wall-foundation contacts.

The model employs simplifying assumptions, such as rigid walls, a flat bottom, and friction-only interactions. Although these choices naturally restrict direct quantitative generalizability to all silo designs (e.g., those with hoppers, very flexible walls, or cohesive materials), they were crucial for establishing a controlled numerical baseline. The results provide a fundamental stress state and a hierarchy of parameter influence, serving as a benchmark for methodically evaluating the impacts on parameter complexity in future studies.

The parametric analysis (Figs. 8, 9, 10, 11, 12, 13, 14, 15, 16 and 17) revealed notable tendencies, explicable by the basic principles of granular materials:

- The little effect of Young’s modulus (E) is a significant result. The observable impact just in the lower regions indicates that wall pressure is mostly determined by stress equilibrium rather than the stress-dependent elastic deformation of the granular assembly. Upon yielding and changing into a plastic state (as described by Mohr-Coulomb), the material’s stiffness significantly influences the resultant stress distribution. The significant effect at the base is probably attributed to a more complex, restricted deformation condition approaching failure.
- The significant positive connection with Poisson’s ratio (v) comes directly from the Poisson effect. A higher ν indicates a greater tendency for the granular material to expand laterally under vertical compression. This produces enhanced pressure on the confining silo wall, a relationship mathematically expressed by the coefficient of lateral earth pressure at rest, K₀ = ν/(1 - ν). The non-linear characteristics of this equation explain the disproportionately significant rise in lateral pressure corresponding to incremental increases in ν, particularly at the base where confining stresses are maximum.
- The linear correlation with material density (ρ) is dictated by the theory of geostatic stress. The vertical stress at a given depth is expressed as σ_v_ = ρ * g * h. Lateral pressure (S11) is dependent upon vertical pressure (often expressed as S11 = K₀*ρ*g*h); hence, each increase in density correspondingly elevates the lateral pressure on the silo wall.
- The adverse correlation with the grain-wall frictional coefficient (µ) confirms the superiority of the Janssen arching mechanism. Increased friction amplifies shear stress activation at the wall contact. This enables a larger percentage of the weight of the stored material to be sent vertically to the silo wall via shear, instead of being exerted horizontally as pressure. This shear transfer significantly reduces the horizontal tension on the wall.
- The minimal impact of cohesion (c) and the angle of repose (φ_r_) with the effect of the internal friction angle (φ) suggests that under confining pressures inside a full silo, the frictional strength of the granular material governs its behavior. Cohesive strength becomes insignificant, and arching efficiency attains its peak at a moderate φ, rendering further increases insignificant for pressure reduction.

The geometry of the silo significantly influences which physical mechanism governs pressure distribution (Figs. 14, 15 and 16):

- Slender silos (h/a ≥ 7.5) exhibit asymptotic pressure profiles well predicted by Janssen’s theory, in which arching is fully established, limiting pressure escalation with height.
- Squat silos (h/a ≤ 1.5) have linear pressure distributions that approach Rankine active earth pressure (S11 = Ka·γ·h), due to their design preventing complete arching development. The system works similarly to a conventional retaining wall. The assumption of slender-silo design is incorrect^7^.

These results have clear implications for design and failure processes. The elevated, concentrated bending moments resulting from the unequal pressure distribution in rigid walls (greater in the center, reduced at the corners) may lead the wall to buckling. Therefore, applying design approaches for thin silos (Janssen) to squat silos would lead to an incorrect underestimation of bending stresses, potentially resulting in structural collapse.

In conclusion, effective silo design requires a focus on the essential parameters (v, ρ, µ) and a thorough understanding of the governing mechanics—arching in slender silos and lateral earth pressure in squat silos—to ensure financial sustainability and structural safety.

## Conclusions

The study aims to develop a model that illustrates the wall-filling pressure in square, flat-bottomed rigid steel silos based on diverse material characteristics and several silo dimensions to enhance structural efficiency. This adaptable model facilitates the analysis of both slender and squat silos, providing an understanding of the pressure distributions across diverse silo geometries. The analysis focused on two main elements: the influence of silo size and the properties of granular materials on wall pressure. The study revealed numerous significant results:

1. Material properties: Lateral wall pressure was shown to be considerably influenced by Young’s modulus, Poisson’s ratio, grain-wall friction coefficient, and internal angle of friction. These parameters significantly affect the lateral wall pressure and the contact of the stored material with the silo walls. However, the wall pressures were only slightly influenced by the cohesiveness of the granular material and the repose angle, suggesting that these factors may be of lesser importance in silo design.
2. Silo dimensions: The study showed that the planform dimensions of the silo significantly affect the pressures exerted on the lateral walls. Therefore, it is not convenient to predict lateral wall pressure of squat silos with slender silos computation methods. In addition, the pressure tends to be linear for squat silos when the plane deformation results as limit are considered.
3. Proportional ratios: Silos with different proportional ratios have nearly the same maximum to lowermost pressure ratios and having nearly the same shape of wall pressure curves. The valuable insights derived from the uniform pressure distribution trends at various sizes will help the establishment of more general design approaches.
4. Practical Implications for Design: The results provide a definitive hierarchy for designers: Poisson’s ratio (v), density (ρ), and wall friction (µ) are essential characteristics that need thorough evaluation, although cohesion (c) and excessive interior friction (φ) may often be ignored for conservative estimations. The selection of the analytical method is fundamentally determined by the slenderness ratio of the silo; Janssen’s theory is suitable for slender silos (h/a ≥ 7.5), whereas a Rankine-type active pressure formulation (S11 = Ka·γ·h) is required for squat silos (h/a ≤ 1.5) to prevent non-conservative and potentially hazardous underestimation of wall pressures and bending moments.

Eventually, these results highlight the need for precise modeling techniques and the complex details of silo design, which provide optimal efficiency and structural integrity in grain storage systems. Rigid-wall models exhibit peak pressure at wall centers, while flexible silos transfer pressure towards corners as a result of wall deformation^7,25^. This variation significantly alters failure mechanisms.

Consequently, optimization of material and silo geometry must include wall flexibility. Design methodologies must distinguish between different kinds of rigidity. Future studies should broaden this framework to examine flexible-walled silos in both static and dynamic conditions, allowing for a comprehensive evaluation of buckling concerns. These findings will facilitate the development of more robust silo designs for agricultural and industrial uses.

## Data Availability

The datasets used and/or analyzed during the current study are available from the corresponding author on reasonable request.
